# Training Syntax to Enhance Theory of Mind in Children with ASD

**DOI:** 10.1007/s10803-022-05507-0

**Published:** 2022-03-31

**Authors:** Stephanie Durrleman, Anamaria Bentea, Andreea Prisecaru, Evelyne Thommen, Hélène Delage

**Affiliations:** 1 Present Address: Department of Medicine, Faculty of Science and Medicine, University of Fribourg, Fribourg, Switzerland; 2 Department of Linguistics, University of Konstanz, Konstanz, Germany; 3 Institute of Psychiatry, Psychology and Neuroscience (IOPPN), King’s College London, London, UK; 4 HETSL, University of Applied Sciences and Arts Western Switzerland, Lausanne, Switzerland; 5 Department of Psycholinguistics and Speech-Language Therapy, Faculty of Psychology and Educational Sciences, University of Geneva, Geneva, Switzerland; 6 Department of Linguistics, Faculty of Humanities, University of Geneva, Geneva, Switzerland

**Keywords:** Autism, Theory of Mind, False belief, Training program, Linguistic intervention

## Abstract

Preschool children with neurotypical development (ND) trained on sentential complements (“X thinks/says *that*”) improve their Theory of Mind (ToM) performance. Can complementation training also enhance ToM in children with Autism Spectrum Disorder (ASD)? Thirty-three children with ASD (*Mage* = 8;11) and 20 younger ND peers (*Mage* = 4;3) were trained on sentential complements (4–6 weeks, 2–3 times per week, via the DIRE i-Pad App). Pre-training and post-training comparisons show that (1) training boosted both complementation and ToM performance across groups; (2) improvements remained 4–6 weeks after training ended; (3) participants with milder ASD symptoms made most gains. Training on sentential complements thus seems beneficial for addressing ToM difficulties in children with ASD, especially those with milder symptoms.

**Supplementary Information:**

The online version contains supplementary material available at 10.1007/s10803-022-05507-0.

The ability to reflect upon others’ mental states, realize that these may differ from one’s own, and consequently understand and predict behaviours, is referred to as Theory of Mind (ToM). Children with Autism Spectrum Disorder (ASD)^1^ are known to display core ToM difficulties (Baron-Cohen, 2000), and investigating a new remediation program for these difficulties is the aim of the current work. It has been suggested that while both *social-perceptive* and *social-cognitive* routes may be relied upon for inferring mental states, people with autism may primarily capitalize on the latter mediated by language (Eigsti & Irvine, 2021; Tager-Flusberg, 2001). Thus, linguistic training could prove beneficial for enhancing ToM in ASD.

The litmus task for assessing ToM is the false-belief (FB) task, where protagonists hold an inaccurate belief about an object either because (a) it was displaced in their absence, or (b) it has a misleading appearance. An example of the first type of FB assessment, also known as the ‘‘Change of Location Task’’ (Baron-Cohen et al., 1985; Wimmer & Perner, 1983) is the Sally-Anne task (Baron-Cohen et al., 1985) where one character (Sally) places a ball inside a basket and then leaves the room. Another character (Anne) then moves the ball to a box and leaves the scene. When Sally returns, participants are asked where she will look for her ball. To answer successfully, participants must acknowledge that Sally holds an inaccurate mental representation about the location of the ball, different from their own, and which will guide her to search for the ball in an erroneous location (the basket). It is well-known that children with autism struggle to grasp that someone may not know what they know, and thus frequently provide reality responses rather than a response reflecting Sally’s subjective (and mistaken) view of reality (Yirmiya et al., 1998). An illustration of the second type of FB task (i.e., involving misleading appearances) is the ‘‘Unexpected Contents task’’ (Gopnik & Astington, 1988), which requires establishing a dual identity for an object by distinguishing between appearance (what it looks like) and reality (what it really is). An example is the ‘‘Smarties task’’: participants are shown a tube of Smarties and asked what they think is inside. After the likely response ‘‘Smarties’’, the tube is opened to reveal some unexpected contents (e.g., pencils), at which point participants are asked to predict what someone else thinks is inside the tube. Children on the autism spectrum commit errors on such tasks, again suggesting an inability to differentiate between subjective and objective perceptions (Baron-Cohen, 1989).

Children with neurotypical development (ND) succeed at tasks such as these around 4–5 years of age (Wellman et al., 2001), showing that they have reached a certain level of mind-reading sophistication (Dennett, 1978), including several key ToM processes like grasping the diversity of mental states (i.e., differentiating between one’s own and those of others), acknowledging that these states are subjective and can include false representations of reality, as well as understanding that such states drive people’s behaviour. In contrast, this important milestone in ToM development is delayed in children with ASD, who exhibit persistent developmental challenges in this sphere, even when their mental age reaches 9 years (Baron-Cohen et al., 1985; Yirmiya et al., 1998). This delay in ToM development could underlie various core social, language, and communication impairments associated with ASD (Frith, 2012; Frith & Frith, 2012; Senju, 2013; Thommen et al., 2011).

## The Relation Between Language and Theory of Mind

Despite ToM impairments being a core difficulty in ASD, a subset of children with this condition, that is, between 20 and 50% (see Baron-Cohen et al., 1985, or Prior et al., 1990) manages to succeed in FB tasks. What allows this subset of children on the spectrum to surmount a central challenge of their condition remains to be determined. It has been claimed that linguistic abilities play a key, mediating role in FB task success. Specifically, children with autism could use language to ‘‘solve theory of mind tasks in an unusually conscious and logical way’’ and do so ‘‘in a verbally-mediated fashion’’ (Happé, 1995: 852–854). In line with this view, it has been suggested that reliance on verbal reasoning for FB attribution could represent a hallmark for individuals with ASD (Bowler, 1992; Durrleman & Delage, 2016; Durrleman & Franck, 2015; Happé, 1995).

A growing body of research provides supporting evidence for the idea that language skills underpin ToM performance, not only in children with ASD, but also in various other clinical populations, such as children with Developmental Language Disorder (DLD, de Villiers et al., 2003; Nilsson & de López, 2016), or deaf children with limited language exposure (Peterson & Siegal, 2000; Schick et al., 2007). Delays in ToM in these populations are contingent on language delays as children potentially lack the linguistic forms and structures needed for creating mental representations about other people’s mind states.

Longitudinal studies, including training studies, also suggest a causal relation between linguistic abilities and mentalizing abilities, with the former influencing the latter. A body of work indeed reveals that in 3 to 5-year-old children with ND, language skills, in particular “complements” such as ‘‘Sally believes/thinks/says *that* the ball is in the basket’’, are predictive of ToM (Astington & Jenkins, 1999; Boeg et al., 2021; de Villiers & Pyers, 2002; Ebert, 2015; Hale & Tager-Flusberg, 2003; Kaltefleiter et al., 2021; Lohmann & Tomasello, 2003; Shuliang et al., 2014). Sentential complements (sentences introduced by the complementizer “that’’) arguably serve as ideal tools for the representation of subjective truths because they show someone else’s (e.g., Sally’s) point of view (de Villiers, 2007, 2021). This could explain why complementation skills rank above other linguistic abilities, such as global syntax or receptive vocabulary, in predicting ToM performance, as revealed in a meta-analysis conducted by Milligan et al. (2007).

While mental-state talk clearly enhances ToM development (e.g., Ebert et al., 2017; Lohmann & Tomasello, 2003), training studies that do not specifically include mental-state verbs (e.g., *think, believe*) have also been effective at promoting ToM reasoning in ND children (Hale & Tager-Flusberg, 2003; Lohmann & Tomasello, 2003; Shuliang et al., 2014), in children with DLD (Durrleman & Delage, 2020; Durrleman et al., 2019), and in those who are deaf or hard-of-hearing (Durrleman et al., 2021), providing sentential complements are targeted. Indeed, Hale and Tager-Flusberg (2003) trained 3 to 5-year-old ND children on complements of communication verbs like ‘‘Sally *says* that (…)’’, with protagonists reporting events erroneously. This training increased participants’ scores on a series of ToM tests, suggesting that the acquisition of these sentential complements contributes to the development of ToM in pre-schoolers. The specific interest of this structure for ToM was further highlighted by the fact that training on a structure of similar syntactic complexity (i.e., relative clauses), did not show any repercussions on ToM (Hale & Tager-Flusberg, 2003). Similarly, Shuliang et al. (2014) showed that training complements of communication verbs gives rise to even higher ToM gains compared to training complements of mental state in ND children aged 3 to 4, possibly because these allow children to directly observe that people’s statements, and thus thoughts, may not coincide with reality. The fact that children trained on complements without mental-state semantics boost their FB task performance suggests that the crucial linguistic ability of complementation itself, rather than the semantics of mental state talk, can suffice to provide a scaffold by which children develop a way of grasping what others think.

It is important to note that the two above-mentioned studies involved erroneous/deceptive reporting of events (i.e., protagonists saying things which were not true), either because they made a genuine mistake (e.g., erroneously perceived something as something else) or lied (knowingly reported something inaccurately). A critical question thus arises, namely whether complementation could facilitate ToM in and of itself, without being coupled with deception. In order to disentangle effects of complementation and deception, Lohmann and Tomasello (2003) as well as Shuliang et al. (2014) have revealed that complementation training in ND children *without* the use of deceptive scenarios also gives rise to improvements in ToM. In contrast, training on deceptive scenarios *without language* does *not* yield ToM gains. More specifically, complementation training involving only the reporting of true events can increase performance on ToM tasks, while training that involves people touching a ‘‘deceptive object’’ (e.g., a sponge that looks like a rock) yet only miming surprise or making minimal exclamations (e.g., “Oh! Look!”), does not increase ToM task performance. These results thus further highlight the interest of complementation independently of deception. Still, the most useful training for consolidating ToM in studies with ND participants have involved *both* complements and deceptive scenarios, suggesting the interest in highlighting both when seeking to enhance mind-reading (Lohmann & Tomasello, 2003). This dual emphasis is the format which has been subsequently successfully incorporated in training studies involving clinical populations, and more specifically 30 children with DLD (mean age = 7;3), and 21 deaf and hard-of-hearing children (mean age = 8;11), yielding significant gains in both complements and ToM measured via different FB tasks (Durrleman & Delage, 2020; Durrleman et al., 2021).

Together, these findings suggest that children’s mastery of sentential complements, in particular of communication verbs, can play a key role in the development of ToM understanding, especially when one highlights the contradiction between what is reported and what can be observed in reality. Given the interest of improving complementation for promoting ToM, it becomes conceivable that a population with specific ToM deficits such as children with ASD may benefit from the training of complements that carefully capitalizes on communication complements and deceptive scenarios. Such training could provide children on the autism spectrum with a strategy to bootstrap their mental state reasoning. No large-scale study has explored the relevance of complementation for ToM in ASD, however various studies have indicated a privileged link between these constructions in this population (Durrleman & Delage, 2016; Durrleman & Franck, 2015; Lind & Bowler, 2009) and one longitudinal study revealed that knowledge of sentential complements of communication verbs significantly predicted ToM performance one year later in 5 to 14-year-old participants with ASD (Tager-Flusberg & Joseph, 2005). This pattern of results suggests that children whose language skills were more advanced by incorporating sentential complements in natural communication were able to use this linguistic knowledge to succeed in tasks that required a representational understanding of the mind. While this body of work provides a theoretical rationale for the role of complements in ToM in individuals with autism, it does not assess the possibility that training on sentential complements could act as a clinical intervention that improves their ToM. One study (Paynter & Peterson, 2013) which trained 24 children with ASD (mean age = 7;0) on the concept of beliefs using thought-bubbles seems to suggest that this population can improve ToM with an intervention which incorporated complements of verbs of cognition (e.g., *think*). Despite complements not being explicitly interpreted by the authors as a key ingredient responsible for the ToM gains observed, the questions and explanations involved in their program incorporated various instances where complements were centre-stage.

The only training specifically targeting complements with children with ASD (aged 5–11) is a pilot study involving a digital ToM training program (in the form of an App administered via iPads; Durrleman et al., 2019). This intervention encouragingly yielded significant post-training gains in FB scores, however it represents only a starting point, as its main aim was to assess if complementation training provided the *most* effective route towards improving FB relative to a control training focused on lexical enrichment. Although this study was done with two small groups of children with ASD aged 5 to 11 (6 per training protocol), post-training improvements only arose in children presented with the complementation protocol and were absent from those trained with the lexical enrichment protocol. These findings seem to indicate the specific interest of complementation compared to a more general linguistic training focused on global vocabulary.

## The Current Study

In light of the preliminary validation of the training protocol (Durrleman et al., 2019; see also Durrleman & Delage, 2020, for children with DLD), the current study examines whether the benefits of complementation training on ToM can be replicated with a larger sample of children with ASD, and further investigates what particular characteristics of the children’s profiles may allow them to benefit best from such a program. Building on the idea that training on sentential complements, in particular those of communication verbs, coupled with deceptive scenarios and pictorial material, may serve to facilitate ToM reasoning, the current training protocol includes all of these essential features.^2^

In order to investigate if training complements of communication verbs boosts complements and facilitates ToM reasoning both in pre-school ND children, and in older children with autism, we compare pre-training and post-training ToM scores in these populations. We assess whether the training program significantly improved performance on complements (*direct effect*) and ToM (*indirect effect*). Given previous findings for training on sentential complements in ND children and in children with DLD, we expect children with ASD, along with their younger ND peers, to benefit from our protocol and enhance both complementation and ToM skills at post-training assessments relative to pre-training assessments. We additionally explore to what extent benefits on ToM emerge similarly across different FB assessments. With this in mind, we administer not only classical verbal FB tasks, following the basic Sally-Anne format (Baron-Cohen et al., 1985), but also low-verbal ones, inspired by a format initially used with deaf children (Woolfe et al., 2002). Such tasks help to shed light on whether complementation can boost FB performance beyond the widely-used, highly verbal FB tasks, and thus suggest a link between complementation and FB reasoning (Durrleman & Delage, 2016).

The study also addresses the issue of whether the hypothesized improvements in complements and ToM can persist over time in children with ASD (*long-term effect*), by re-testing these children 4–6 weeks after training ceased. We predicted that the effects detected during immediate post-testing will be maintained at these delayed post-tests. Finally, the study seeks to shed light on the *individual differences* that can potentially influence the effect of training in our clinical group, such as non-verbal IQ and severity of autism spectrum symptomatology. In sum, our three research questions are:

(1) Can children with ASD, as well as younger ND peers, benefit from training on complements to boost not only complements but also ToM, assessed both verbally and low-verbally?

(2) Can gains in complements and ToM be maintained over time in ASD, and more specifically 4–6 weeks after training has ceased?

(3) What abilities allow certain children on the autism spectrum to benefit more from the training protocol?

## Methods

### Participants

Participants included 33 children with ASD (30 boys, *Mage* = 8;11, *SD* = 27 months, age range 5;7 to 14;9) and 20 children with ND (8 boys, *Mage* = 4;3, *SD* = 8 months, age range 3;0 to 6;0). Differences in age were due to the fact that difficulties on ToM have been attested at different phases of development in these populations. Participants were recruited in Geneva, Lausanne and Paris, from specialised schools for the group with ASD, and from kindergartens or day-care facilities for those with ND. All were native French-speakers.^3^ Children with ASD were required to have received a diagnosis by a qualified professional using gold standard tools: the DSM-5 (American Psychiatric Association, 1994), the Autism Diagnostic Observation Schedule/ADOS (Lord et al., 2003; translated by Rogé et al., 2015) or the Autism Diagnostic Interview/ADI-R (Rutter et al., 2003; translated by Rogé et al., 2011). The ND children had no history of language, social or behavioural difficulties and had to be attending kindergartens or day-care facilities without support. All participants also had to score below 70% on comprehension of ToM and complements, in order to allow a margin of progression in these domains, which were targeted by the training program. Finally, the children’s language comprehension level had to allow them to understand simple subject-verb-object sentences, which was essential in order to benefit from the training included in the current study. This had to initially be indicated by parents, and then confirmed by experimenters using the Exalang scale, a standardized test common in French (Helloin & Thibault, 2006).

This study was approved by the Ethics Committee of the Faculty of Psychology and Educational Sciences of the University of Geneva as well as from the Geneva Cantonal Ethics Commission and was also declared at “La Commission Nationale de l’Informatique et des Libertés (CNIL)” in France. Parents provided written informed consent for participation.

### Procedure

**Fig. 1:**
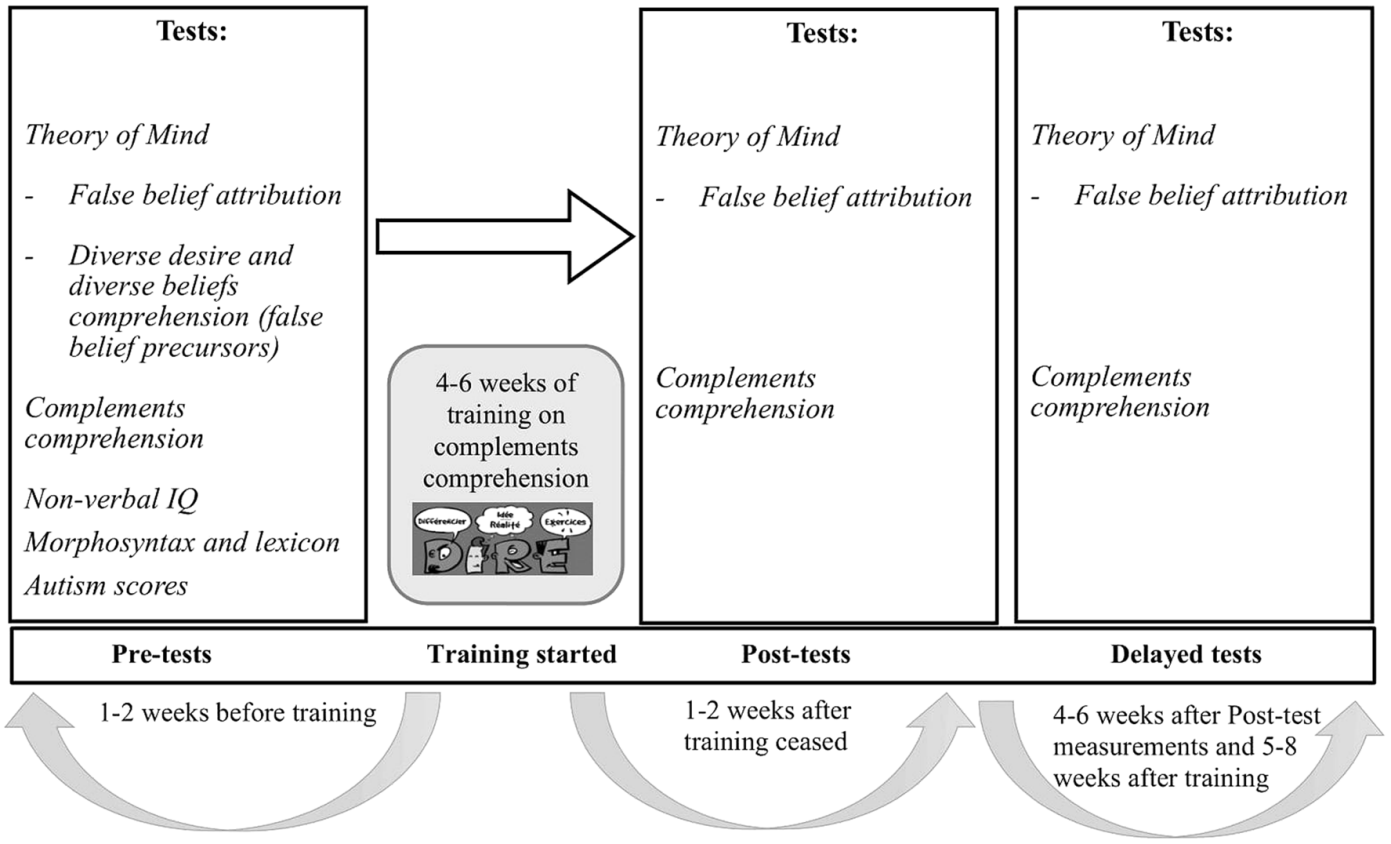
Measurements conducted at each assessment point

Participants were assessed on ToM and complementation at three time points: ‘‘pre-(training) test’’ (1–2 weeks before training), ‘‘post(-training) test’’ (1–2 weeks after training) and ‘‘delayed (post-training) test’’ (4–6 weeks after training, and at least 4 weeks after immediate post-training tests). We chose to administer follow-up/delayed tests 4–6 weeks after the end of training, because this way the break between immediate post-tests and delayed post-tests is similar to that between pre- and immediate post-tests (since the duration of the training protocol was also 4–6 weeks). In this way, the effects of training were observed in a longitudinal fashion. Figure 1 illustrates the study design and shows that measurements of ToM (FB) and Complements Comprehension were conducted at all time points.

The participants performed these tests on computers, contrary to the training, which involved iPads. The reason for this clear distinction between testing and training was to ensure that any differences in scores obtained between pre- and post-tests could not be attributed to gains in dexterity with the actual test materials. Similarly, the stories/scenarios generated by the DIRE application during training differed from those in the tests. By carefully ensuring that children never saw any training items that were the same as those in the testing, improvements between pre-test and post-test scores could not be explained by similarities between testing and training materials. Finally, the items of the three test batteries (pre-tests, immediate post-tests and follow-up-tests) all differed from one another, thus boosts in test scores could not be due to similarities between testing materials.

### Tests

#### Theory of Mind Tests

ToM was assessed using tasks of belief attribution. FB tasks ensure that participants have a mature ToM, because in order to attribute a false belief, they have to grasp that others’ beliefs are representations of reality based on which we can predict behaviour (Dennett, 1978). We used two types of tasks (*verbal* and *low-verbal*) and each task included 6 FB items interspersed with 6 true belief (TB) items, yielding a total of 24 items (12 FB and 12 TB). TB items were not measures of ToM, because a reality response sufficed to answer correctly, but were included as fillers to diversify the material and ensure that participants could modulate their predictions depending on varying circumstances (Forgeot d’Arc & Ramus, 2011).

**Fig. 2:**
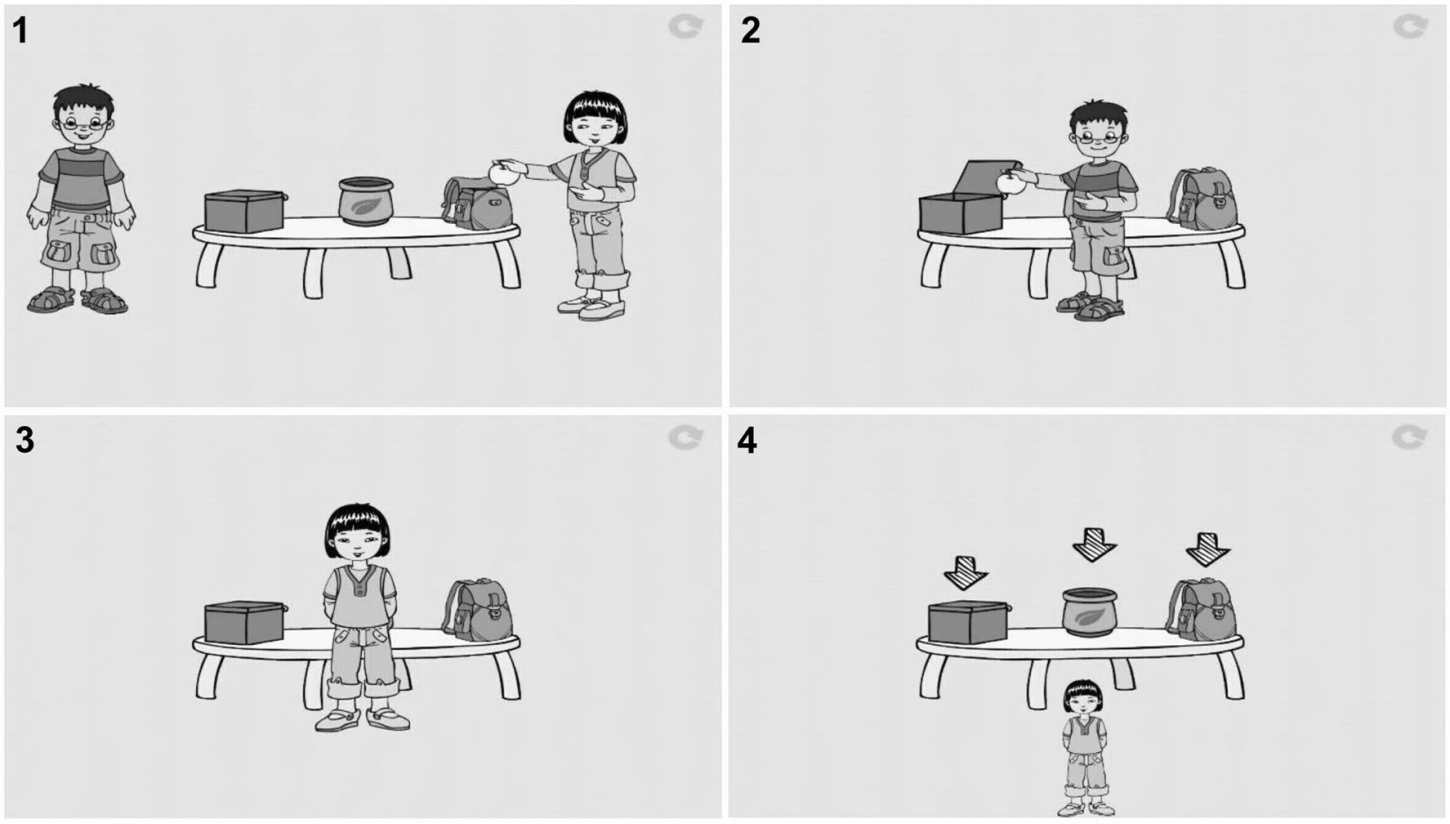
Illustration of a verbal Theory of Mind task

The *Verbal FB task* involved animated scenarios on a computer, accompanied by an anecdote (inspired by Baron-Cohen et al., 1985). In this task, Protagonist 1 places an object in Location 1 and leaves the scene. Then Protagonist 2 moves this object to Location 2. When Protagonist 1 returns, unaware of the object’s change of location, participants have to predict where s/he would search for the object. To respond successfully, children must differentiate between their mental state (their knowledge that the object is in Location 2) and that of Protagonist 1 (the false belief that the object is still in Location 1). Figure 2 illustrates such an item. FB items were interspersed with TB items that don’t require making attributions about mental states since the objects are either not displaced or displaced by Protagonist 2 in front of Protagonist 1. In these instances, children simply had to point to where the object was really located.

**Fig. 3:**
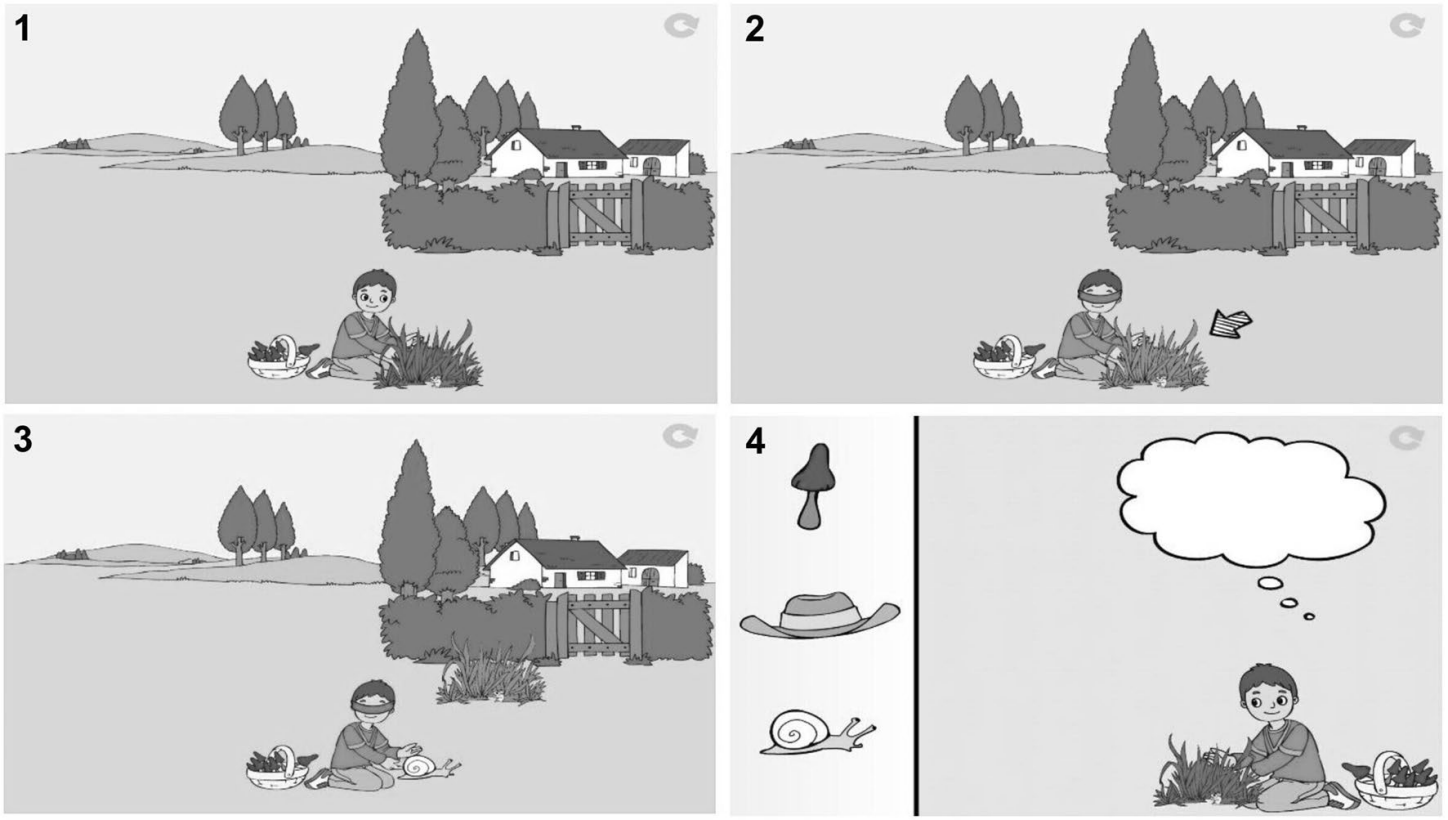
Illustration of a low-verbal Theory of Mind task

The *Low-Verbal FB task* minimized linguistic demands (along the lines of Woolfe et al., 2002), allowing to obtain a pure measure of ToM without linguistic confounds. Animated images provided the information needed to interpret the scenario and were coupled with a very short narrative, always involving a character unable to see an object s/he was trying to grasp. Figure 3 exemplifies such a scenario, where a boy, who is picking mushrooms, at one point becomes unable to see what he is reaching for, in this case because there is a clump of grass hiding it. In the TB condition, this object would continue to be a mushroom, while in the critical FB condition, this would be something else (e.g., a snail). The corresponding narrative would simply comment what could already be observed from the images, i.e. “A boy is picking mushrooms, but now his hands are hidden by a clump of grass. Click to see what is there!” The child would click on the screen and see a snail appear under the clump of grass, and then they had to indicate, by selecting one answer out of three options, what the character thinks he grasped. In order to answer the FB item correctly, children had to acknowledge that the character’s belief was different from reality (i.e., that the object was a mushroom rather than a snail).

In addition to FB tasks, at the first testing session we also administered a concise assessment of certain ToM precursors, namely diverse desires and diverse beliefs. Indeed, as explained in the introduction, children’s understanding of mental states is not monolithically composed of FB but rather follows a developmental trajectory between the ages of 3 to 5 composed of various critical steps, such that certain forms of mental state understanding succeed or follow each other (Wellman & Liu, 2004). More specifically, before the age of 4, children become aware that people can have *diverse desires* before they become aware that people can have *diverse beliefs* (see Wellman & Woolley, 1990). Thus, we wanted to take a measure of ToM precursors to see if previous consolidation of these notions also played a role on children’s ability to benefit from the training to progress to FB reasoning. The tests of precursors to FB attribution, inspired by Burnel et al. (2018), were all low-verbal and included 3 items of diverse desires and 3 of diverse beliefs.

#### Complements Comprehension Test

For sentential complements, children watched animated videos narrated by pre-recorded stories in which a character either erroneously reports an event (false complement) or accurately reports an event (true complement), as in de Villiers and Pyers (2002). This task included a total of 12 items (6 false complements and 6 true complements). Children were required to report the content of the complement, so the protagonist’s reported event, and thus the complements always involved communication verbs. As a result, children were not required to “read” the character’s mind, as they simply had to recall the sentential complement. For instance, they heard: “The grandmother asks the dad what Victor is doing. And the dad answers that Victor is picking apples in the garden.” Sometimes this is what Victor is seen doing and sometimes he is doing another activity, and then children were asked: “What did the dad say that Victor is doing?”.

#### Standardized Tests

In addition to assessments of ToM and complements, at the first testing session only, children also completed (i) the Raven’s Coloured Progressive Matrices (RPM) Test (Raven et al., 1998) as a measure of non-verbal reasoning abilities; (ii) the Exalang 3–6 scale (Helloin & Thibault, 2006) for linguistic (receptive lexical skills, morphosyntactic skills, narrative production) and non-linguistic skills (memory, assessed via repetition of numbers and monosyllabic words, and attention, assessed via identification of a specific word when heard in a list); (iii) for the children with ASD, the Childhood Autism Rating Scale/CARS (Schopler et al., 1988) to assess the severity of ASD symptoms, as we did not have access to the ADOS and ADI-R scores due to medical confidentiality. Online Appendix A gives the measures and scoring for the CARS scale, as well as cut-off points for the three groups (mild/moderate/severe).

#### Training

After completing the pre-tests, children underwent a training phase where exercises from the program DIRE (Durrleman et al., 2019) were administered 2 to 3 times a week on tablets. The training contains a total of 100 different training items and was spread over approximately four uninterrupted weeks so that each child did between 8 and 12 training sessions lasting 30–40 min each. If experimenters observed that children were still struggling with tasks at this point, participants again saw the training material a second time maximum. The DIRE training was previously assessed in other studies—for example, in a study by Durrleman and Delage (2020) with 30 children with DLD. DIRE is the French verb meaning ‘‘to say’’ and its program focuses on training complements of communication verbs, steering clear of complements of mental state verbs for the reasons outlined in the introduction. More specifically, the training included sentences like ‘‘Character X says that Y’’, because complements of communication verbs (e.g., ‘‘says’’) have been validated in previous studies (Hale & Tager-Flusberg, 2003; Lohmann & Tomasello, 2003; Shuliang et al., 2014) as an effective tool for representing subjective truths. In addition, DIRE is an acronym for ‘‘Differentiating Ideas from Reality via Exercises”, which was our ultimate goal with training complementation in this study, given the specific difficulties in mentalizing experienced by children with ASD. The DIRE protocol involved *five types of activities* in which participants first watched short animated videos and then had to either select the correct image/character that matched the target sentence, or to repeat a sentence given by the experimenter (see Online Appendix B for illustrations). All the activities targeted complements. The first activity (Activity 1) included *infinitival complements*, while all other activities (Activity 2 through 5) focused on *tensed complements*, which are those thought to specifically support ToM (de Villiers, 2007). We wanted to ensure that children mastered complements with an infinitival verb before targeting tensed complements because infinitival complements are the first form of complements to emerge in child speech and they provide the foundation on which tensed complements are consolidated in language development (Bloom et al., 1989; Diessel, 2004). Training was administered via iPads, thus circumventing various social deficits associated with ASD (APA, 2013), and capitalizing on the finding that screen-based tools have proven efficacy with clinical populations (Alzrayer et al., 2014).

## Results

The children were first assessed using various linguistic and cognitive standardized measures, described in the Methods section above and administered at pre-test. While there were no significant differences between the two groups on most measures, including on ToM precursors, there were significant differences on some measures, as revealed by a Welch *t* test (Online Appendix C, Table C1). These significant differences were related to age, auditory attention and non-verbal reasoning. The scores indicate that children with ASD are significantly older than ND children (*t*(39) = 10.79, *p* < .001), as expected given that children had to show FB difficulties to participate in the study, and these persist later in autism. Significant differences also emerge in raw scores for auditory attention (*t*(43) = 3.36, *p* = .001) and non-verbal reasoning (*t*(47) = 3.73, *p* < .001), showing that children with ASD, who are also older, display higher attentional capacities and non-verbal intelligence than the ND group. However, if we consider children’s age-normalized percentile scores for non-verbal reasoning (*t*(33) = − 3.52, *p* = .001), the scores of the ASD group were associated with lower percentile points than those of the ND group.

ND and ASD children were also tested on true complements and true beliefs (verbal and low-verbal), which served as indicators of attention to the task. The results (Online Appendix C, Table C2) indicate that the ASD group performs similarly to the younger ND group at pre-test on true complements and on verbal and low-verbal TB items. This was confirmed by the statistical analysis (see following section for details) showing that ND and ASD children did not differ on these measures (*β* = − 0.319, *SE* = 0.556, *z* = − 0.573, *p* = .57).

### Statistical Analysis

Our dependent variable was response accuracy to the false items across three conditions (Complements, verbal ToM, and low-verbal ToM). As the dependent variable had a binary outcome (success or failure in a single trial), we used generalized linear mixed effects regression models (Jaeger, 2008). The analyses were conducted using the *glmer* function of the *lme4* package in *R* (Bates et al., 2015; R Core Team, 2019).

We analysed the data in two steps. The first analysis (addressing Question 1) compared the effect of training between the two groups and included *Group* (ND, ASD), *Test Time* (PreTest, PostTest), and *Condition* (False complements, verbal FB, low-verbal FB) as fixed predictors, as well as their interactions. Random intercepts were included for *Subjects* and *Items*, as well as random slopes by Subject for *Test Time* and *Condition*.

The second analysis (addressing Questions 2 and 3) focused on the ASD group only in order to establish whether the effect of training persists through time by retesting children after a delay of 4–6 weeks after training ceased. The fixed factors were *Test Time* (PreTest, PostTest, DelayedTest), and *Condition* (False complements, verbal FB, low-verbal FB), as well as their interaction. This analysis also included (i) the CARS scores, to examine to what extent the degree of ASD symptoms impacts the effect of training; (ii) the Raven’s scores, to determine to what degree non-verbal reasoning contributed to the effect of training, both at post-test and at delayed test. Both scores were included as continuous variables in the model. The maximal random effect structure supported by the data for the second analysis included only intercepts for *Subjects*. Below we report only the statistically significant results (see Tables D1 and D2 in Online Appendix D for the full models).

Question 1: Can children with ASD, as well as younger ND peers, benefit from training on complements to boost not only complements but also ToM?

**Fig. 4:**
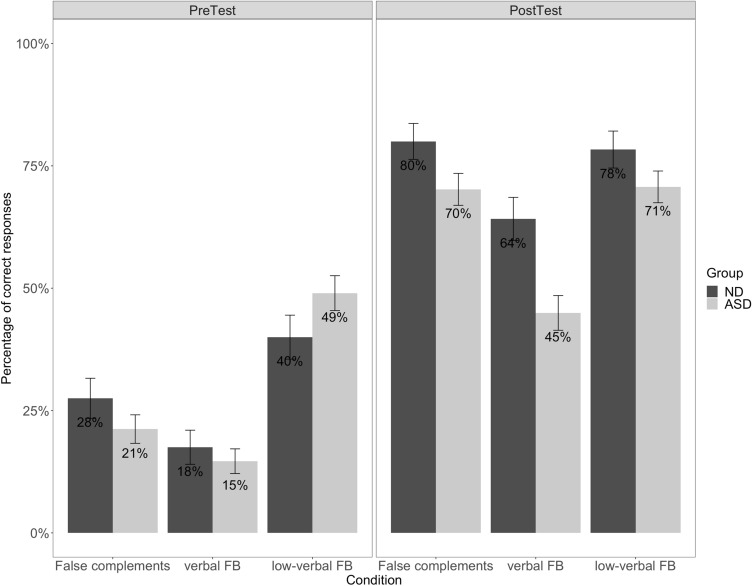
Correct responses by condition at pre-test and post-test for ND and ASD children

The results (Fig. 4) show comparable performance between the two groups at pre-test when tested on false complements, as well as on verbal and low-verbal FBs. Although response accuracy improved at post-test in both groups, the ND children show overall higher performance at post-test than the children with ASD (see the analysis below for a more detailed explanation of the difference in performance between ND and ASD children).

The analysis (see Online Appendix D) revealed a significant main effect of *Test Time* (*β* = 2.519, *SE* = 0.294, *z* = 8.544, *p* < .001), indicating that both ND and ASD children gave more correct answers at post-test than at pre-test. The significant main effect of *Condition* shows that overall children gave more correct responses to verbal FBs compared to low-verbal FBs (*β* = − 1.673, *SE* = 0.323, *z* = − 5.174, *p* < .001) and to low-verbal FBs than to false complements (*β* = 0.495, *SE* = 0.241, *z* = 2.046, *p* = .04). The *Group* × *Test Time* interaction approaches significance (*β* = 1.078, *SE* = 0.565, *z* = 1.905, *p* = .05). Subsequent pairwise comparisons show that this is due to the absence of a significant difference between the ND and ASD children’s responses at pre-test (*β* = 0.038, *SE* = 0.275, *z* = 0.139, *p* = .89), while at post-test the ND children were significantly more accurate across conditions than the children with autism (*β* = 1.116, *SE* = 0.545, *z* = 2.047, *p* = .04). The significant interaction *Test Time* × *Condition* (*β* = − 0.808, *SE* = 0.362, *z* = − 2.227, *p* = .02) reveals that response accuracy for false complements was significantly lower than for low-verbal FBs at pre-test (*β* = − 0.899, *SE* = 0.272, *z* = − 3.308, *p* = .002), while the two conditions did not differ significantly at post-test (*β* = − 0.091, *SE* = 0.330, *z* = − 0.276, *p* = .96).

Question 2: Can children with ASD maintain gains in complements and ToM over time?

**Fig. 5:**
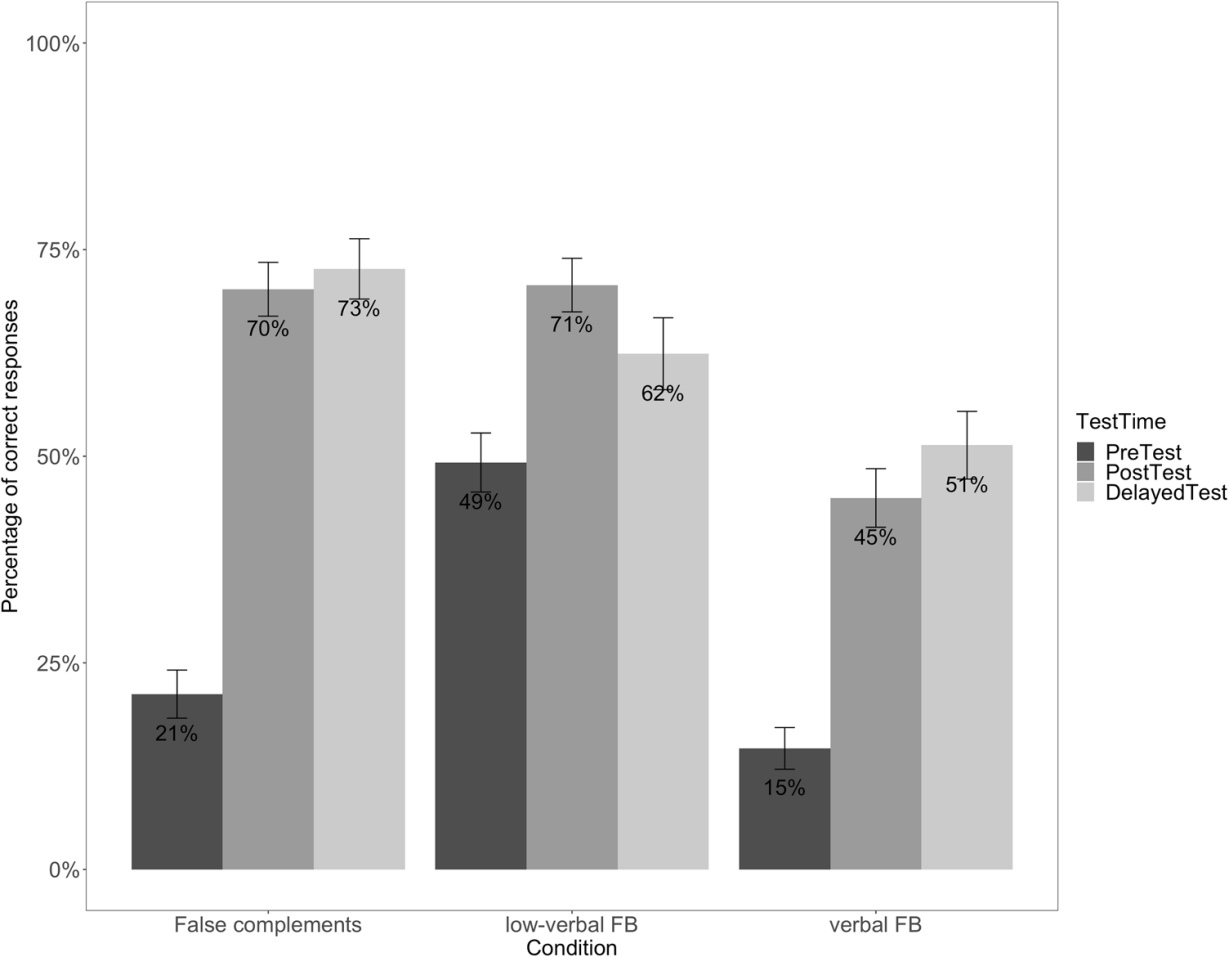
Correct responses by condition at pre-test, post-test and delayed test for ASD children only

Figure 5 summarizes the results of the ASD group^4^ for each condition across all three testing times (pre-test, post-test, delayed test) and shows that response accuracy for the low-verbal FB condition is higher at post-test and delayed test than at pre-test.

The statistical analysis (see Online Appendix D, Table D2) revealed significant interactions between *Test Time* × *Condition*. Together with subsequent pairwise comparisons, these indicate that (i) there is no difference in comprehension between false complements and verbal FBs at pre-test (*β* = 0.536, *SE* = 0.280, *z* = 1.916, *p* = .13), but false complements are comprehended significantly better than verbal FBs at post-test (*β* = 1.338, *SE* = 0.240, *z* = 5.567, *p* < .001); (ii) low-verbal FBs receive more correct responses than verbal FBs at post-test (*β* = 1.316, *SE* = 0.239, *z* = 5.509, *p* < .001), but there is no difference between the two conditions at delayed test (*β* = 0.440, *SE* = 0.272, *z* = 1.621, *p* = .24).

Question 3: What abilities allow certain children with ASD to maximally benefit more from the training protocol?

We also examined the contribution of autism severity, as indicated by CARS scores, and of non-verbal reasoning, as measured by Ravens scores, to the training effect. CARS and Ravens scores were included as continuous variables^5^ in the analysis (see Online Appendix D, Table D2). The interaction *Test Time* × *CARS* shows a strong tendency towards significance (*β* = − 0.037, *SE* = 0.019, *z* = − 1.956, *p* = .050) and the interaction *Test Time* × *Ravens* reaches significance (*β* = 0.101, *SE* = 0.022, *z* = 4.470, *p* < .001). This indicates that children’s *CARS* scores and their performance in the non-verbal reasoning task modulate response accuracy. The results of a post-hoc analysis aimed at disentangling the effect of *CARS* and *Ravens* on response accuracy at the various *Test Times* revealed a significant effect of *CARS* at pre-test (*β* = 0.032, *SE* = 0.016, *z* = 1.974, *p* = .048), but not at post-test.

**Fig. 6:**
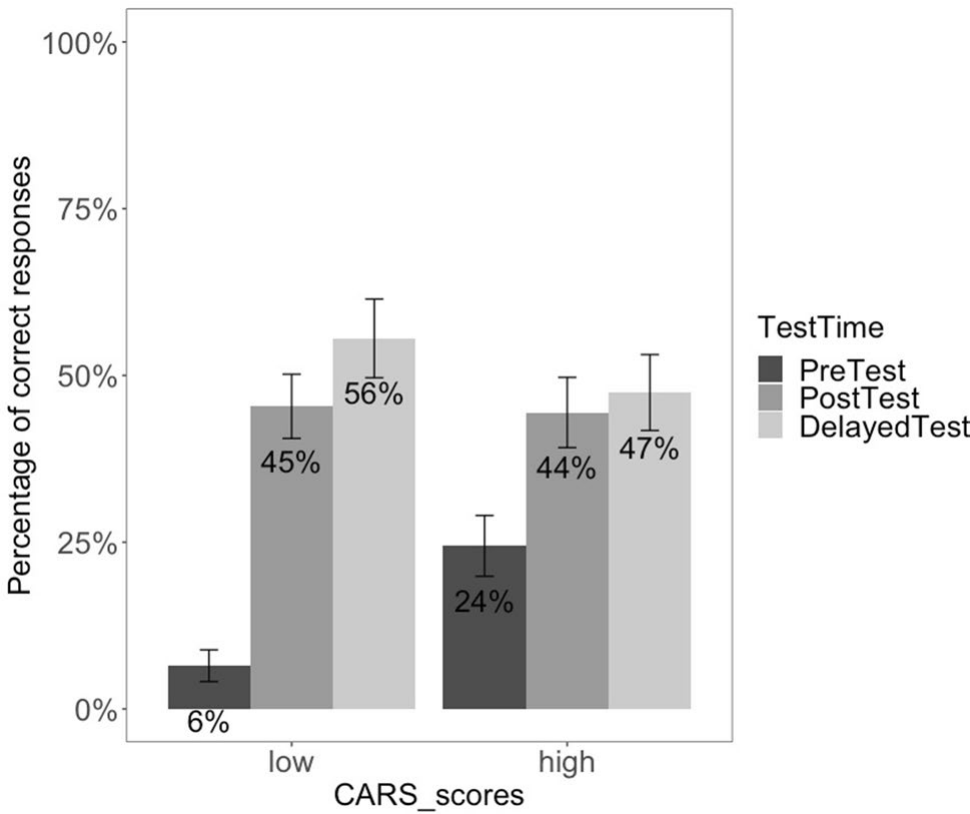
Correct responses by CARS scores at pre-test, post-test and delayed test for the verbal FB condition

This difference seems to be driven by children’s comprehension of verbal FBs: children with lower CARS scores (range 15–30) showed greater increase in response accuracy (39%) between pre-test and post-test than children with higher CARS scores (range 31–47), whose response accuracy only increased by 20% (see also Fig. 6, where we report the accuracy scores for the verbal FB items by CARS group).

**Fig. 7:**
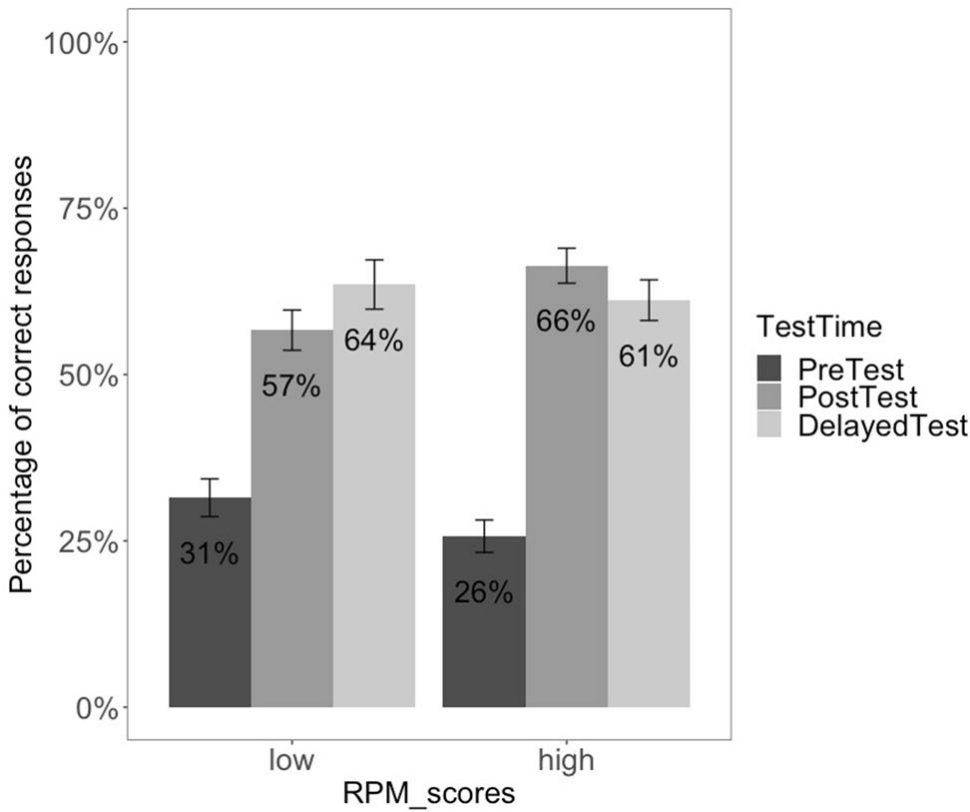
Correct responses by non-verbal reasoning (RPM) scores at pre-test, post-test and delayed test

The post-hoc analysis also revealed a significant effect of *Ravens* only at Post-test (*β* = 0.075, *SE* = 0.034, *z* = 2.197, *p* = .03), thus the overall effect of training at Post-test is higher for children with higher non-verbal reasoning abilities (see also Fig. 7, which indicates the overall response accuracy for the ASD participants divided into high and low groups, according to their non-verbal reasoning scores^6^). Finally, no effect of CARS or *Ravens* emerges at Delayed Test.

## Discussion

This longitudinal study explored the extent to which a training protocol targeting sentential complements of communication verbs (e.g., X says *that*) improves performance on complements and ToM in ND children and in children with ASD, a group displaying ToM impairments. The current work also explored which traits increase the efficacy of this intervention. We thus addressed three main research questions:

*Question 1: Can children with ASD, as well as their younger ND peers, benefit from training on complements to boost not only complements but also ToM? Are ToM boosts observable in different types of FB tasks (verbal, low-verbal)?* Complementation training *does give rise* to gains in ToM in both ND children and children with autism. Our findings reveal that mastery of complementation improves FB understanding in these populations, since participants of this study obtained higher scores in immediate post-training compared to pre-training tests assessing both complements and FB, thus suggesting that the training protocol generates significant improvements in these abilities.

ToM boosts were observed in FB reasoning, and encouragingly emerged not only with a highly verbal task, but also with a low-verbal assessment. This indicates that children trained on complementation improve their FB across a variety of testing situations. Amongst the three types of tests administered (for false complements, low-verbal FB and verbal FB), the highest increase in performance occurred for the complementation tasks, from 21% before training to over 70% accuracy at post-training tests. This could suggest that children exhibit the largest improvements in the linguistic domain of complements via a direct effect of training. Their improved skills for complementation are subsequently transferred to the ToM domain, so that children’s increased mastery of complements indirectly facilitates their ToM task performance. Children on the autism spectrum appear to use language as a tool to succeed at mental-state reasoning, such that an intervention targeting subordinate clauses allows them to grasp mental representations (e.g., thoughts) more successfully.

Among the ToM tests, children were more accurate in the low-verbal task compared to the high verbal one (at the post-training test). A possible explanation for increased performance in the low-verbal task is that this type of assessment is more accessible for children with ASD. This task kept language requirements minimal by being highly visual and allowed children to follow the situations just by looking at the images and applying their belief attribution. In contrast, the verbal task involved a longer story and higher computational load associated with the tracking of an object manipulated by two different people and with retaining a verbal anecdote in working memory, which could be a potential source of difficulty in and of itself, over and beyond ToM challenges (Durrleman & Delage, 2016; Vyshedskiy et al., 2017).

*Question 2: Can gains in complements and ToM be maintained over time in children with ASD, and more specifically, will they still arise in delayed post-tests, administered 4–6 weeks after training has ceased?* The improvements observed *did persist* over time, as performance was similar between immediate and delayed post-tests. A previous meta-analysis by Hofmann et al. (2016) revealed that most ToM studies assessed training effects in a very short time frame (max. 13 days), suggesting that more research was needed to probe the long-term efficacy of training protocols. To address this limitation, our current study thus provided a longitudinal investigation where post-training effects were measured at delayed time-points (4–6 weeks after training).

*Question 3: What abilities allow certain children with ASD to maximally benefit from the training protocol?* After assessing the effects of training as a clinical intervention supporting ToM development, our study examined the contribution of ASD severity (assessed via CARS) and non-verbal abstract reasoning (measured by Raven’s matrices). Our pattern of results suggests that a significant relation exists between the severity of ASD symptoms and test performance: children with less severe ASD symptoms (measured before the first training session) showed greater gains in response accuracy between pre- and post-test relative to children with more pronounced ASD symptoms.^7^ This could reflect the fact that the DIRE training protocol works best for the subgroup of children with milder cases of autism, suggesting that this group has better treatment outcomes compared to their peers with more severe ASD symptomatology. Similarly, the overall effect of training was mediated by non-verbal reasoning abilities, such that children with higher non-verbal skills performed better in post-training tests, pointing to the fact that overall higher functioning contributes to better gains from this intervention.

Following the significant improvements in ToM reasoning observed here, results support the idea that sentential training could be used as a clinical intervention for children with ASD who are yet to consolidate first-order FB reasoning. A next step could be to check whether training on recursive sentential complements (*X says that Y claims that Z*) can take children on the spectrum to even more sophisticated levels of FB reasoning (i.e., of the second-order). Preliminary work suggests this may indeed be the case (Polyanskaya et al., 2021), although as the authors themselves underline, confirmation of this is pending a large-scale intervention study that would also include follow-up (delayed) testing.

Given that success in FB reasoning is associated with success in day-to-day social behaviours (Astington, 2003; Astington & Edward, 2010; Astington & Jenkins, 1999; Astington & Pelletier, 2005; Derksen et al., 2018; Mazza et al., 2017), interventions focusing on improving FB reasoning hold the promise of a genuine and crucial change in children’s social cognition, an area specifically affected by the autistic condition (Baron-Cohen, 1995; Baron-Cohen et al., 1985). The increased FB reasoning observed in our current study may thus help to attenuate core social difficulties in ASD. Future studies should seek to uncover to what extent this is indeed the case by specifically measuring if higher FB scores translate to real-life changes, with parental questionnaires such as the Parent-Report Measure of Assessing Individual Differences in Children’s Theories of Mind (Tahiroglu et al., 2014).

In sum, the improvements observed in children with ASD participating in the training program *DIRE* suggest that it could be promising to include this program as a clinical intervention for linguistic and cognitive difficulties attested in ASD. Given that ToM abilities underpin a variety of social skills which are specifically affected by ASD, future work should seek to assess whether cascading benefits arise in daily social contexts, in particular in the subset of children with milder autism symptoms and higher reasoning abilities, who seem particularly suited for the DIRE program.

## Supplementary Information

Below is the link to the electronic supplementary material.

Supplementary file1 (DOCX 27 kb)

## Footnotes

1 This work takes into account the fact that people with ASD do not only express preference for being referred to this way, but also as ‘autistic’ and ‘people on the autism spectrum’, ‘persons with autism’, etc. (see Kenny et al., 2016, Bottema-Beutel et al., 2021). We thus use these terms interchangeably.

2 Indeed the main aim of this work being to assist children with ASD to improve ToM, rather than to determine the individual impacts of each of these factors, all were combined to allow for optimal efficacy with a clinical population showing specific challenges in this domain.

3 Specific data on race/ethnicity were not recorded, however a gross measure of socioeconomic status (SES) was collected and its results are reported in footnote 5.

4 Seven children were no longer available for retesting at Delayed Test. We therefore only included the responses of the remaining 26 children for this comparison. We agree with one anonymous reviewer that it would be interesting to explore the potential long-term impact of this training in ND children as well. However, only a small number of the initial 20 ND children assessed at pre- and post-test were available for retesting at Delayed Test. We will aim to address this in future work.

5 We also took a coarse measure of socio-economic status (SES) by considering the mother’s level of education. We checked the impact of SES on the gains obtained from training at Post-Test and Delayed Test. Stepwise model comparison showed that adding SES (coded as *university degree* or *no university degree*) did not improve the model (*p* = .755). Therefore, we did not include it in the analysis reported here. We also checked for the impact of ToM precursors on children’s ability to benefit from the training to progress to FB reasoning. This effect was, however, not significant (*β* = -0.027, *SE* = 0.066, *z* = -0.420, *p* = .674).

6 We created the two groups by calculating the median of the Ravens scores for the ASD group. The values in the lower half of the data sample were included in the *low RPM_scores group*, while those in the higher half of the data sample were included in the *high RPM_scores group*.

7 Interestingly, children with less pronounced ASD symptoms, as reflected by lower CARS scores, were also younger, which could explain why they also obtained lower scores at pre-test than their older peers with more pronounced ASD symptoms. Nevertheless, the younger, milder cases catch up at post-test, thus appear to benefit more from training than their older peers with more pronounced ASD traits.
