# Supplementary material for: Training Syntax to Enhance Theory of Mind in Children with ASD
Source: J Autism Dev Disord. 2022 Mar 31;53(6):2444–57. doi: 10.1007/s10803-022-05507-0 (PMC10229475; doi:10.1007/s10803-022-05507-0)
Supplement: Supplementary file 1 — Supplementary file1 (DOCX 27 kb) [file 10803_2022_5507_MOESM1_ESM.docx]

**Appendices**

**Appendix A**

**The Childhood Autism Rating Scale – items and cut-off points for diagnosis**

| *Domains assessing behaviours associated with Autism* |
| --- |
| 1. Relating to people. |
| 1. Imitation, social-emotional understanding. |
| 1. Emotional response, emotional expression and regulation of emotions. |
| 1. Body use. |
| 1. Object use, object use in play. |
| 1. Adaptation to change, adaptation to change/restricted interests. |
| 1. Visual response. |
| 1. Listening response. |
| 1. Taste, smell and touch response and use. |
| 1. Fear or nervousness, fear or anxiety. |
| 1. Verbal communication. |
| 1. Nonverbal communication. |
| 1. Activity level, thinking/cognitive integration skills. |
| 1. Level and consistency of intellectual response. |
| *Impressions assessed by the clinician* |
| 1. General impressions. |

The Childhood Autism Rating Scale (CARS; Schopler et al., 1980, 1988) consists of 15 domains assessed on a 4-point scale (higher scores are associated with more marked autistic traits). Total scores range from 15 to 60 and can indicate *mild-to-moderate* levels of Autism (for scores between 30 to 36.5) or *severe* Autism (for scores from 37 to 60), while scores below 30 indicate that the individual is not in the Autistic range.

**Appendix B**

**Five types of activities involved in the DIRE training protocol**

| Activity 1 | Inspired by the Laureate Learning Systems program (Wilson & Fox, 2013) which specifically taught complements with an infinitival verb, this activity trained the easiest form of embedded sentences with an infinitival verb (‘‘Jill needs *to clean* the bathtub.*’’)* as these are the first complements to emerge in child speech and provide the foundation on which tensed complements are consolidated in language development (Bloom et al., 1989; Diessel, 2004). |
| --- | --- |
| Activity 2 | The child heard a complement with a tensed verb (‘‘The little girl screamed that there was a spider in the bathtub.”) and then had to select the content of the complement (‘‘What did the girl scream?”). The content of the complement either coincided with what was represented on the screen (the girl really sees a spider), or not (she just sees a stain that looks like a spider from a distance). The event reported via the complement was systematically illustrated within speech bubbles, thus children could directly assess whether the complement had an accurate or inaccurate truth-value (i.e., whether it was a true or false complement). We hypothesized that this would allow children to infer that some characters mistakenly reported reality because they had a representation of reality that is subjective (i.e., the girl shouted something that was the result of her perceiving one thing for something else) and often inaccurate. |
| Activity 3 | Children were trained on producing tensed complement sentences. First, they saw animations where characters reported activities they were doing (‘‘Look, I’m petting the dog.’’). In some scenarios this report was accurate, while in others the characters had their eyes closed and reported a false belief (they were in fact petting a cat and not a dog). After checking children’s comprehension of the complement (“What did the girl say?”), the program also asked children if the character was right, and they were furthermore asked to repeat related complements which were freshly modeled for them, accentuating the divergence between reported speech and the state of affairs in the real world (‘‘The girl says that she is petting a dog, but look, it is actually a cat.’’). The experimenter could intervene whenever the child was unable to repeat the sentence, e.g., if it was too long, it was subsequently proposed in two smaller parts (“She says that there is ... . Now look, it is only a … .”). |
| Activity 4 | This activity included two levels of training. First, children had to distinguish between simple sentences without a complement (“There's a fly on the cake.”) and sentences with complements of communication verbs (“The girl shouted that there was a fly on her cake.”) by selecting the accurate image amongst two options (*the fly actually on the cake* or *the girl with a speech bubble showing a fly on a cake*). Second, they were asked to differentiate between two complements by selecting the image best capturing a character’s description of a situation, such as: ‘‘The girl shouted that there was a fly on her cake.’’ and ‘‘The boy shouted that there was a snake under his bed.’’. In these instances, the child had to identify ‘‘who said what’’. |
| Activity 5 | Children participated in a truth-value game, where they heard a story ending with a complement (“Look, the girl screamed there was a spider / a stain in the bathtub.”) and were asked to determine if the content of the complement / what the character reported was right or not. |

**Appendix C**

**Table C1. Mean scores (and standard deviation) for standardised measures at pre-test for ND (neurotypical development) children and children with ASD (Autistic Spectrum Disorder)**

|  | ***ND*** | ***ASD*** | ***TD/ASD comparison*** |  |
| --- | --- | --- | --- | --- |
| *Age (months)* | 50.9 (8.3) | 107 (27.3) | ***t*(39) = 10.79, *p <* .0001** |  |
| *Receptive lexicon (/60)* | 55.0 (5.3) | 54 (4.5) | *t*(35) = -0.68, *p* = .497 |  |
| *Receptive morphosyntax (/15)* | 10.1 (2.3) | 10.1 (2.8) | *t*(46) = -0.05, *p* = .955 |  |
| *Narrative comprehension (/12)* | 6.3 (3.3) | 5.4 (3.2) | *t*(40) = -1.08, *p* = .286 |  |
| *Short-term memory (/8)* | 6.0 (1.8) | 6.8 (1.09) | *t*(27) = 1.95, *p* = .060 |  |
| *Phonological working memory (/12)* | 10.3 (3.2) | 9.8 (2.3) | *t*(31) = -0.55, *p* = .582 |  |
| *Auditory attention (/20)* | 9.6 (5.3) | 14.8 (5.8) | ***t*(43) = 3.36, *p =* .001** |  |
| *Non-verbal reasoning (raw scores) (/36)*  *Non-verbal reasoning (percentiles)*  *ToM Precursors (/6)* | 14.2 (3.5) | 19.7 (7.1) | ***t*(47) = 3.73, *p =* .0005** | |
|  | 45 (25) | 21.7 (17.3) | ***t*(33) = -3.52, *p =* .001** | |
|  | 4.8 (1.4) | 4.5 (1.8) | *t*(43) = -0.52, *p* = .600 | |

**Appendix D**

**Table D1. Estimated odds ratio, SE, and associated p-values of main and interaction effects for the ND versus ASD comparison**

| ***Generalised linear mixed model***  ***Accuracy ~ 1 + Group * Test Time * Condition + (1 + Test Time + Condition \| Subject) + (1 \| Item)*** | | | | |
| --- | --- | --- | --- | --- |
| *Fixed effects* | Estimate | SE | z-value | p-value |
| *Intercept* | 0.027 | 0.167 | 0.164 | .869 |
| *Group _NDvs ASD_* | 0.577 | 0.326 | 1.770 | .076 |
| *Test Time_PostTest vs PreTest_* | 2.519 | 0.294 | 8.544 | **<.001***** |
| *Condition_low-verbal FB vs False Complements_* | 0.495 | 0.241 | 2.046 | **.040*** |
| *Condition_verbal FB vs low-verbal FB_* | -1.673 | 0.323 | -5.174 | **<.001***** |
| *Group_ND vs ASD_ : Test Time_PostTest vs PreTest_* | 1.078 | 0.565 | 1.905 | **.056.** |
| *Group_ND vs ASD_ : Condition_low-verbal FB vs False complements_* | -0.426 | 0.458 | -0.931 | .352 |
| *Group_ND vs ASD_ : Condition_verbal FB vs low-verbal FB_* | 0.558 | 0.621 | 0.898 | .369 |
| *Test Time_PostTest vs PreTest_ : Condition_low-verbal FB vs False Complements_* | -0.808 | 0.362 | -2.227 | **.025*** |
| *Test Time_PostTest vs PreTest_ : Condition_verbal FB vs low-verbal FB_* | 0.460 | 0.402 | 1.144 | .252 |
| *Group_ND vs ASD_ : Test Time_PostTest vs PreTest_ :*  *Condition_low-verbal FB vs False Complements_* | 0.758 | 0.654 | 1.160 | .245 |
| *Group_ND vs ASD_ : Test Time_PostTest vs PreTest_ :*  *Condition_verbal FB vs low-verbal FB_* | -0.179 | 0.723 | -0.248 | .804 |

*Note.* The fixed factors in the two analyses were coded using repeated contrasts, which test consecutive factor levels against each other (Schad et al., 2019) and the continuous variables were centred around the mean. The goodness-of-fit of alternative models was assessed by comparing the Akaike information criterion scores (Akaike, 1974). A decrease of at least 2 in the Akaike information criterion scores means that the inclusion of a factor significantly improves the goodness-of-fit of the model. As the models fitted were rather complex, we also used an optimising function (the *bobyqa* optimizer) within the *glmer* function in *R* in order to ensure that we obtain a stable model which does not give any convergence errors (Linck & Cunnings, 2015).

**Appendix D**

**Table D2. Estimated odds ratio, SE, and associated p-values of main and interaction effects for the ASD group only**

| ***Generalised linear mixed model***  ***Accuracy ~ 1 + TestTime*Condition + TestTime*CARS + TestTime*Ravens + Condition*CARS + Condition*Ravens + (1 \| Participant)*** | | | | |
| --- | --- | --- | --- | --- |
| *Fixed effects* | Estimate | SE | z-value | p-value |
| *Intercept* | 0.024 | 0.151 | 0.165 | .869 |
| *Test Time_PostTest vs PreTest_* | 1.808 | 0.151 | 11.953 | **<.001***** |
| *Test Time_DelayedTest vs PostTest_* | -0.156 | 0.155 | -1.010 | .312 |
| *Condition_verbal FB vs False complements_* | -0.935 | 0.152 | -6.127 | **<.001***** |
| *Condition _low-verbal FB vs verbal FB_* | 1.203 | 0.150 | 7.999 | **<.001***** |
| *CARS* | -0.125 | 0.018 | 0.671 | .502 |
| *Ravens* | -0.010 | 0.021 | 0.458 | .647 |
| *Test Time_PostTest vs PreTest_ : Condition_verbal FB vs False complements_* | -0.801 | 0.368 | -2.176 | **.029*** |
| *Test Time_DelayedTest vs PostTest_ : Condition_verbal FB vs False Complements_* | 0.405 | 0.359 | 1.127 | .259 |
| *Test Time_PostTest vs PreTest_ : Condition _low-verbal FB vs verbal FB_* | -0.559 | 0.356 | -1.570 | .116 |
| *Test Time_DelayedTest vs PostTest_ : Condition _low-verbal FB vs verbal FB_* | -0.875 | 0.362 | -2.147 | **.015*** |
| *Test Time_PostTest vs PreTest_ : CARS* | -0.372 | 0.019 | -1.956 | **.050.** |
| *Test Time_DelayedTest vs PostTest_ : CARS* | -0.323 | 0.018 | -0.002 | .998 |
| *Test Time_PostTest vs PreTest_ : Ravens* | 0.101 | 0.022 | 4.470 | **<.001***** |
| *Test Time_DelayedTest vs PostTest_ : Ravens* | -0.623 | 0.021 | -2.847 | **<.01**** |
| *Condition_verbal FB vs False complements_ : CARS* | 0.033 | 0.018 | 0.178 | .858 |
| *Condition _low-verbal FB vs verbal FB_ : CARS* | 0.032 | 0.018 | 1.770 | .076 |
| *Condition_verbal FB vs False complements_ : Ravens* | -0.054 | 0.022 | -0.024 | .980 |
| *Condition _low-verbal FB vs verbal FB_ : Ravens* | -0.038 | 0.022 | -1.744 | .081 |
